# Streptococcus agalactiae Endocarditis Involving the Tricuspid Valve and Device Leads in a Patient With an Automated Implantable Cardioverter-Defibrillator (AICD)

**DOI:** 10.7759/cureus.97566

**Published:** 2025-11-23

**Authors:** Mehrdad Zarghami, Anubhav Agarwal, Alexandria Jaramillo, Grace m Pena

**Affiliations:** 1 Internal Medicine, Jamaica Hospital Medical Center, New York City, USA; 2 Family Medicine, Jamaica Hospital Medical Center, New York City, USA; 3 Pulmonary and Critical Care Medicine, Jamaica Hospital Medical Center, New York City, USA

**Keywords:** aicd, device-related infection, septic emboli, streptococcus agalactiae, tricuspid valve endocarditis

## Abstract

Infective endocarditis involving cardiac device leads remains a serious complication in patients with intracardiac hardware, particularly in the setting of bacteremia. We present a case of a 47-year-old female with heart failure with reduced ejection fraction (HFrEF) and an automated implantable cardioverter-defibrillator (AICD) who developed Group B *Streptococcus *(*Streptococcus agalactiae*) bacteremia with evidence of both lead-associated and tricuspid valve vegetations. The case highlights diagnostic challenges in transthoracic echocardiography (TTE), multidisciplinary management, and the role of early device extraction in such cases.

## Introduction

Infective endocarditis (IE) is a life-threatening condition characterized by infection of the endocardial surface of the heart, most commonly involving the cardiac valves. It may also affect prosthetic material, such as prosthetic valves or cardiac implantable electronic devices (CIEDs), including pacemakers and defibrillators. IE typically presents with a constellation of clinical features such as persistent fever, bacteremia, new or changing heart murmurs, and embolic phenomena. The incidence of IE has increased over the past two decades, partly due to the growing use of intracardiac devices, hemodialysis, and invasive procedures in aging and comorbid populations [1].

The most common pathogens associated with IE vary depending on the host and clinical context. In native valve IE, *Staphylococcus aureus *is now the leading pathogen in many developed countries, followed by viridans group streptococci, enterococci, and coagulase-negative staphylococci [2]. *Staphylococcus aureus *is also the most common cause of CIED-related infections due to its high affinity for device surfaces and its ability to form biofilms. Fungal pathogens, HACEK organisms (*Haemophilus*, *Aggregatibacter*, *Cardiobacterium*, *Eikenella*, and *Kingella* species), and Gram-negative bacilli are less common but important in specific patient populations [3].

Group B *Streptococcus *(*Streptococcus agalactiae*) (GBS) is a beta-hemolytic, Gram-positive organism that is well known for causing neonatal sepsis, meningitis, and infections in pregnant women. However, in recent years, there has been an increasing recognition of invasive GBS infections in nonpregnant adults, particularly among those with chronic illnesses such as diabetes mellitus, malignancy, liver disease, and heart failure [4,5]. In adults, GBS can lead to skin and soft tissue infections, bacteremia, osteomyelitis, and, more rarely, infective endocarditis. When GBS causes IE, the infection is often aggressive, with large vegetations, high rates of systemic embolization, and considerable morbidity and mortality [6].

GBS endocarditis involving intracardiac devices is extremely rare, and the combination of tricuspid valve and device lead vegetations, as in our patient, is particularly uncommon; in such right-sided infections, pulmonary and septic emboli represent central manifestations causing respiratory failure, highlighting the need for prompt recognition and intervention.

## Case presentation

A 47-year-old female with heart failure with reduced ejection fraction (HFrEF) (EF 30-35%) status following automated implantable cardioverter-defibrillator (AICD) placement, type 2 diabetes mellitus, and chronic anemia presented with a one-month history of fever, exertional dyspnea, and right-flank-radiating low back pain, having visited urgent care and the ED multiple times before admission. On arrival, she was febrile to 102°F and tachycardic (heart rate (HR) 110). Laboratory evaluation revealed leukocytosis (WBC 19.1 x10⁹/L), anemia (hemoglobin (Hb) 7.9 g/dL, hematocrit (Hct) 24.5%), low RBC (3.10 x10¹²/L), low mean corpuscular volume (MCV) (79.1 FL), and elevated red cell distribution width (RDW) (25.1%) (Table 1). Blood cultures grew *Streptococcus agalactiae *(Group B *Streptococcus*). Imaging demonstrated bilateral pulmonary emboli and multiple septic emboli (Video 1), as well as vegetations involving the AICD lead (Video 2) and the tricuspid valve (Video 3), which together shaped both the diagnostic approach and management strategy. She was started on heparin and broad-spectrum antibiotics, including ceftriaxone and vancomycin, plus gentamicin. After culture sensitivity was obtained, she was de-escalated to ceftriaxone IV daily, with lead extraction surgery subsequently performed.

**Table 1:** Laboratory results with the reference range MCV: mean corpuscular volume; RDW: red cell distribution width

Parameter	Patient Value	Normal Reference Range
WBC (×10⁹/L)	19.1	4.0 – 10.0
Hemoglobin (g/dL)	7.9	Female: 12.0 – 16.0
Hematocrit (%)	24.5	Female: 36 – 46
RBC (×10¹²/L)	3.10	4.2 – 5.4
MCV (fL)	79.1	80 – 100
RDW (%)	25.1	11.5 – 14.5
Blood Culture	*Streptococcus agalactiae *(Group B *Streptococcus*)	Negative (no growth)

**Video 1:** Bilateral pulmonary emboli and multiple septic emboli

**Video 2:** Vegetations involving the AICD lead AICD: automated implantable cardioverter-defibrillator; TTE: transthoracic echocardiography

**Video 3:** Vegetations involving the tricuspid valve TTE: transthoracic echocardiography

## Discussion

This case highlights a rare but serious presentation of *Streptococcus agalactiae *(Group B *Streptococcus*, GBS) endocarditis involving both the tricuspid valve and an automated implantable cardioverter-defibrillator (AICD) lead. While GBS is a well-known cause of neonatal sepsis and peripartum infections, its role in adult infective endocarditis (IE) remains infrequent, accounting for a small proportion of IE cases in nonpregnant adults. Even more rare is its association with cardiac implantable electronic device (CIED) infections, which are typically dominated by *Staphylococcus aureus *and coagulase-negative *Staphylococcus *species

This case illustrates an uncommon presentation of GBS endocarditis in a patient with an AICD. Although GBS is a rare cause of CIED-associated infections, when identified, the risk of embolization, valve destruction, and systemic sepsis necessitates prompt management. TTE remains an initial imaging modality, though transesophageal echocardiography (TEE) may be required for better visualization. Combined antimicrobial therapy and early surgical or percutaneous device removal are the cornerstones of therapy. The presence of septic pulmonary emboli further emphasizes the importance of rapid diagnosis and intervention.

Pulmonary embolism (PE) in the setting of right-sided infective endocarditis may arise both from bland thrombus formation and from septic embolization of vegetations. Septic emboli can lead to pulmonary infarcts, cavitary lesions, and progressive parenchymal destruction, distinguishing them from isolated thrombotic PE [7,8]. In our case, the co-existence of acute bilateral pulmonary emboli and multiple septic emboli underscores the severe embolic potential of tricuspid valve and lead-associated vegetations. The recognition of this dual process is critical, as management requires both anticoagulation for thrombotic PE and prolonged antimicrobial therapy for septic emboli, along with definitive source control through device extraction [9].

The presence of large tricuspid and lead vegetations in this patient raised immediate concern for device-related infective endocarditis. Although *Streptococcus agalactiae *(GBS) is an uncommon cause of endocarditis in adults, its identification in both blood cultures and lead tip cultures supports a definitive diagnosis. The involvement of the right-sided valve and intracardiac device, along with evidence of septic pulmonary emboli, highlights a classic but infrequent presentation of lead-associated endocarditis [10,11].

Imaging played a critical role in delineating the extent of infection. The TTE revealed a large mobile vegetation on the tricuspid valve, while the cardiac CT scan confirmed multiple peripheral emboli in the pulmonary arteries, consistent with septic embolization [12]. Although TTE provided initial diagnostic insight, TEE is generally considered superior for characterizing intracardiac device infections and would have provided additional resolution of the vegetations and lead involvement [13]. In this case, the initial TTE and clinical picture were sufficiently compelling to prompt early transfer for advanced management.

The size of the vegetations, exceeding 2 cm on the tricuspid valve and 1.5 cm on the ICD lead, is clinically relevant. Vegetation size has been associated with increased risk of embolization and poorer outcomes [14] and is one of the criteria considered when deciding on surgical versus percutaneous extraction. Although surgical valve intervention is rarely required for isolated right-sided endocarditis, prompt lead and device removal are essential for source control in cases of confirmed device infection [15]. Delays in extraction are associated with increased morbidity, persistent bacteremia, and relapse risk [16].

Our patient was hemodynamically stable and afebrile at presentation but had persistently positive blood cultures before transfer. The clinical team initiated ceftriaxone, a beta-lactam with efficacy against GBS, in line with susceptibility data [17]. The addition of aminoglycoside therapy is sometimes considered for synergy, particularly early in the course, although the benefit in GBS endocarditis is not clearly established [18]. 

This case highlights key considerations in managing cardiac device-related infections, particularly when caused by less common organisms. It also emphasizes the importance of early imaging, prompt microbiologic diagnosis, and adherence to established protocols for device removal and antibiotic therapy. The multidisciplinary decision to proceed with device extraction was appropriate and aligned with current consensus guidelines [3]. Ongoing follow-up will be important to monitor for relapse and guide future discussions on device re-implantation.

## Conclusions

In patients with unexplained fever and intracardiac devices, especially with bacteremia, clinicians should maintain high suspicion for CIED-related endocarditis, as pulmonary embolism and septic emboli represent core manifestations; early multidisciplinary management, targeted antibiotics, and timely device extraction are crucial, and these embolic events carry diagnostic and prognostic significance equal to the endocarditis itself.

## References

[1] Cahill TJ, Baddour LM, Prendergast BD (2016). Infective endocarditis. Lancet.

[2] Murdoch DR, Corey GR, Hoen B (2009). Clinical presentation, etiology, and outcome of infective endocarditis in the 21st century: the International Collaboration on Endocarditis-Prospective Cohort Study. Arch Intern Med.

[3] Baddour LM, Wilson WR, Bayer AS (2015). Infective endocarditis in adults: diagnosis, antimicrobial therapy, and management of complications: a scientific statement for healthcare professionals from the American Heart Association. Circulation.

[4] Skoff TH, Farley MM, Petit S (2009). Increasing burden of invasive group B streptococcal disease in nonpregnant adults, 1990-2007. Clin Infect Dis.

[5] Sendi P, Johansson L, Norrby-Teglund A (2008). Invasive group B Streptococcal disease in non-pregnant adults : a review with emphasis on skin and soft-tissue infections. Infection.

[6] Sambola A, Miro JM, Tornos MP (2002). Streptococcus agalactiae infective endocarditis: analysis of 30 cases and review of the literature, 1962-1998. Clin Infect Dis.

[7] Samman A, Babi A (2025). Pulmonary septic emboli following endocarditis: a case report. Open J Clin Diagn.

[8] Orhun N, Rajab I, Ekin U, Horani G, Ismail M (2025). Unveiling rare pulmonary complications in infective endocarditis: pneumatoceles and pneumothorax in a case series with contextual literature review. J Investig Med High Impact Case Rep.

[9] Comeaux S, Jamison K, Voeltz M (2023). Contemporary features and management of endocarditis. Diagnostics (Basel).

[10] Baddour LM, Epstein AE, Erickson CC (2010). Update on cardiovascular implantable electronic device infections and their management: a scientific statement from the American Heart Association. Circulation.

[11] LE KY, Sohail MR, Friedman PA (2011). Clinical predictors of cardiovascular implantable electronic device-related infective endocarditis. Pacing Clin Electrophysiol.

[12] Habib G, Lancellotti P, Antunes MJ (2015). 2015 ESC Guidelines for the management of infective endocarditis: the task force for the management of infective endocarditis of the European Society of Cardiology (ESC). Endorsed by: European Association for Cardio-Thoracic Surgery (EACTS), the European Association of Nuclear Medicine (EANM). Eur Heart J.

[13] Boyle S, Arvanitaki A, Oldfield K (2025). A multimodality imaging approach to the diagnosis of infective endocarditis: incremental value of combining modalities. Int J Cardiovasc Imaging.

[14] Chu VH, Cabell CH, Benjamin DK Jr (2004). Early predictors of in-hospital death in infective endocarditis. Circulation.

[15] Sandoe JA, Barlow G, Chambers JB (2015). Guidelines for the diagnosis, prevention and management of implantable cardiac electronic device infection. Report of a joint Working Party project on behalf of the British Society for Antimicrobial Chemotherapy (BSAC, host organization), British Heart Rhythm Society (BHRS), British Cardiovascular Society (BCS), British Heart Valve Society (BHVS) and British Society for Echocardiography (BSE). J Antimicrob Chemother.

[16] Sohail MR, Uslan DZ, Khan AH (2007). Management and outcome of permanent pacemaker and implantable cardioverter-defibrillator infections. J Am Coll Cardiol.

[17] Baddour LM, Wilson WR, Bayer AS (2005). Infective endocarditis: diagnosis, antimicrobial therapy, and management of complications: a statement for healthcare professionals from the Committee on Rheumatic Fever, Endocarditis, and Kawasaki Disease, Council on Cardiovascular Disease in the Young, and the Councils on Clinical Cardiology, Stroke, and Cardiovascular Surgery and Anesthesia, American Heart Association: endorsed by the Infectious Diseases Society of America. Circulation.

[18] (2020). Prevention of group B streptococcal early-onset disease in newborns: ACOG committee opinion, number 797. Obstet Gynecol.

